# Strychnine as a Poison—Trial of George W. Green for Murder

**Published:** 1855-02

**Authors:** 


### SELECTIONS.

### *Strychnine as a Poison—Trial of George W. Green for Murder.*

At the December term of the Circuit Court in this city, Judge Morris, presiding, Mr. George W. Green was tried and convicted, on the charge of having murdered his wife, in September last, by giving her Strychnine for that purpose

By the testimony of various witnesses, it was shown that Green and his wife had lived unhappily together for several years; that she died suddenly, and was buried under circumstances, calculated to excite suspicions of crime, and which led to a coroner's inquest. Below, we give so much of the medical testimony, as relates to the Post-mortem appearances of the body of Mrs. Green, and the detection of Strychnine in the contents of her stomach. It should be stated, that owing to some misunderstanding in relation to some portion of the testimony, between the Judge and the council for the prisoner; a new trial has been granted to the latter. That part of the evidence to which we have alluded, is as follows, viz:

*Dr. Max Myers* called and sworn. Resides in this city; am a physician; was at post-mortem examination of Mrs. Green; the body was very much swollen, echimosis on the throat, extending about four inches; eyes swollen; tongue somewhat rigid; did not examine the position of the feet examined the uterus and the stomach; only saw the latter; Dr. Bird opened it; I took the scalpel and found some medicine and an appearance of cheese; the stomach was full or nearly so; from the condition of the stomach, the food had been taken some 12 or 15 hours previous; saw no symptoms of ordinary disease; found a fœtus of four months in the uterus; saw no symptoms of natural death; from what I saw, could not give an opinion as to cause of death. The coroner brought a glazed jar; I told him to wash it carefully; he did so two or three times; I was very particular that it should be clean; it was a pickle jar. Know Dr. Morfit; he has gone to Washington.

By Mr. McIlroy—Discovered no indications of cholera; have had considerable experience in such diseases. No traces of inflammation on the mucous membrane of the stomach.

Cross examined by Mr. Arnold—Had considerable experience in cholera; cholera prevailed extensively and malignantly in this city in September—chances of recovery in a person taken with the disease, very slight.

The examination was on Monday about 1 P. M. Dr. Bird, the Coroner, Drs. Morfit and Freer and myself, were present. Mr. Ravel went with us to show us the house. I merely directed the washing of the jar, it was a glazed earthen one. The spinal chord was not examined; I told Dr. Freer to examine it, because I wished the examination to be thorough. Had she died of strychnine, the spinal chord and brain would give the strongest indications; it produces contractions of the muscles, and acts powerfully on the spinal chord and brain. From the post-mortem could not say positively that strychnine caused her death. It is a remedy used for intermittents and neuralgia. Those diseases prevailed in this city somewhat extensively; it is a common remedy, first introduced by Dr. Brainard; have read the article in the Medical Journal from his pen. Since then it has been considerably used for a medicine

Strychnine and quinine look very much alike. An inexperienced person could not distinguish between them; I myself could not, by means of the *taste* alone. Strychnine taken into the stomach is absorbed very readily. After it has been resolved into its component parts, it may be reproduced.

Direct examination resumed—Strychnine is a very powerful poison. It is not as popular a remedy as formerly; it is usually given in solution, the usual quantity being from 1-12 to 1-8 of a grain. An inexperienced person would not put up 1-8 or 1 10 of a grain with exactness. It is not usual to give out strychnine in quantities of 4 or 5 grains, and leave it discretionary with the patient or family to divide it as they want it. It is sometimes given out, however, for external applications, and inserted in small incisions in the skin. It is as dangerous when thus used, as when used internally.

Cross-examination resumed—Strychnine ceased to become a popular remedy about the time of the publication of the article before alluded to.

(The stomach of the deceased, and its contents, were here sent for.)

*Dr. J. V. Z. Blaney*—Am a practical analytical chemist; have been practicing since 1841; have had considerable practice since that time. I received what I was informed was the stomach of the deceased, Mrs. Green, and its contents, about the 26th or 28th of September last; part of it had the appearance of undigested food; there were also some of the intestines. The undigested contents of the stomach were in a jar, closed with a cork.

The stomach itself was in another similar jar, and the intestines in a third. They were all closed with corks. I made an analysis of the contents of the stomach. In this I was assisted by my brother-in-law, Mr. Butler. By following the process of Professor Stas, of Brussels, I discovered what I was convinced was strychnine. (The doctor here minutely described the process to which he alluded) Suspecting the presence of an alkaloid in the contents of the stomach, I began by adding to a portion of these contents twice its weight of pure and very strong alcohol. I added afterwards 12 to 20 grains of tartaric acid, and introduced the mixture into a flask, and heated it to 150 deg. After it had completely cooled, I filtered it, washed the insoluble residue with strong alcohol, and evaporated the filtered liquid in vacuo. I then dissolved the residue in the smallest possible quantity of water, and introduced the solution into a small test tube, and added little by little of bicarbonate of soda, until the effervescence of carbonic acid ceased. I then agitated the whole with four or five times its bulk of pure ether, and left it to settle. When the ether, swimming on the top, was perfectly clear, the last step consisted in decanting it in a very dry place for spontaneous evaporation.

The evaporation of the ethereal solution left a milky-looking watery fluid, and this was evaporated to perfect dryness. Alcohol was added, which re-dissolved it. The spontaneous evaporation of the alcohol left a residuum of a yellow color, containing *minute crystals*, much contaminated with animal matter. Distilled water was now added, and the crystals washed. The water dissolved the coloring matter, &c., leaving the crystals in a state of purity. I then subjected the crystals to three different tests. I removed several of the crystals upon the point of a penknife, and dropped upon them a few drops of nitric acid. The result was the production of a lemon yellow color. I did not consider this test as entirely characteristic; but proceeded to remove other crystals as before. Upon these I dropped a little sulphuric acid, and added a little of the red prussiate of potash. On stirring this with a glass rod, a magnificent purple color was developed of considerable permanence, and very intense. This gradually passed to a crimson red, then to a scarlet red, which remained permanent for a number of hours. This last test is the one suggested by Dr. E. Davy, of Dublin, and is regarded as characteristic of the presence of strychnine. I treated another portion of the crystals with sulphuric acid, to which was added a drop of a solution of the yellow chromate of potash. In this solution the purple color was equally distinct, but not intense as was produced before, and was evanescent, the purple passing off rapidly, and being succeeded by a lilac color. These tests were made before the Coroner's inquest was held. I have since that time submitted them to other tests. I have examined them under a microscope of high power. By its aid I could perceive that when magnified to eight hundred diameters, they presented the appearance of four sided prisms, that being the form under which strychnine crystalizes. The liquid which drained from the crystals was intensely bitter. The capsule was placed under an air-pump.

The other substances were treated in the same way until I obtained the residuum from the evaporation of ether. The etherial residue, from containing a great deal of animal and coloring matter, would not yield the crystals. It was necessary to use further means to purify the alkaloid. The etherial residue was accordingly treated with dilute sulphuric acid, which would dissolve an alkaloid if present, forming a sulphate solution in water. Part of the coloring matter was thus left behind, and the solution was filtered. To the filtering solution of the sulphate was added pure carbonate of potassa. (Here the results of the analysis—crystals and precipitates—were brought into Court.) After neutralization with potassa; the mixture was introduced into a test tube, strong alcohol added, and the whole agitated. The alcohol was then poured off, and this was continued several times, until the alkaloid set free by the carbonate of potassa, was presumed to be dissolved by the alcohol. A sufficient amount of the carbonate of potassa was added to the sulphate to prevent mixture. This solution was evaporated, and gave a residue still colored, but which I rendered sufficiently pure by washing to enable me to apply the before mentioned tests, two of which, the tests of Davy and Laforte, are regarded as characteristic.

In one case, the washings of alcoholic residuum, after standing a number of days, formed a precipitate which I collected on this small filter, (shows filter,) and to this participate, which was not soluble in water, I applied the tests of Davy and Laforte. The tests acted characteristically. I also applied a further test, recommended in Soubeiran's Pharmacy. To the suspected substance, I added a drop of sulphuric acid, containing from one to five per cent of nitric acid. This was mixed with a glass rod. A minute portion of deutoxide of lead was added; and on mixing these, the same magnificent purple color, of great intensity, was insantly produced, shading away to lilac, red, &c., and more rapidly than in the cases of Davy's test.

The contents of this filter were then submitted to a microscope of a magnifying power of 800 and 1100 diameters. Under the latter, sides and angles showing crystaline forms were clearly discernable. In one of these crystals, I could discern a square figure which I presumed to be the base of a square based octohedron, and in which form, strychnine may crystalize. The portions submitted to the microscopes were treated with a drop of acid solution, in which they were dissolved. On the spontaneous evaporation of this acid solution, fine radiating crystals were produced, of different forms from those exhibited formerly, proving salt formed which is characteristic of vegetable alkalies. I have examined the stomach but have not succeeded in obtaining absolute conviction in regard to the existence of strychnine in the tissue of the stomach. (Exhibits capsules.) The quantity of crystals that I originally produced was sufficient to enable me to apply the three tests that I have mentioned.

The doctor here performed a variety of experiments before the jury, showing them the manner in which he had detected the presence of strychnine in the contents of the stomach. He places on the bottom of a small saucer, a portion of the precipitate collected from the filter. Adds one drop oil of vitrol. Mixes with a glass rod. Adds portion of red prussiate of potash in powder. It changes to a black or purple color. Mixes with a glass rod. Color changes from a crimson to a scarlet red. This is Davy's test.—Laforte's is by the yellow chromate of potash. Detaches crystal from the glass capsule, places it on the saucer. This test is very rapid. The color appears and instantly vanishes. I will use the same test applied to a precipitate. (Test successful.) Have no doubt as to the substance found in the stomach; believe it to be strychnine. It produces death from exciting convulsions, more particularly spasms of the muscles of respiration. Probably through its action on the spinal cord. Cant say whether it has an effect on the coats of the stomach or not. It is absorbed more or less rapidly according to the manner of administration, whether as a solid or in solution. It passes into the circulation. Its effects are the same when injected into a vein, as when taken internally. The portion that was active in producing death, might have been removed from the stomach by absorption. A person could not live many hours after taking a poisonous dose of strychnine. The question, what quantity of strychnine would produce death can only be answered conditionally. It depends on age, sex, sleep, fullness or emptiness of the stomach, and other circumstances. A half grain of the sulphate of strychnine is the smallest dose that I have ever known to produce death. This was the case of Dr. Watson, who died in fourteen minutes. In other cases, death has ensued in an hour and a half from taking three fourths of a grain. There are some external appearances which all physicians agree, are indicative of death by strychnine. Among these effects are rigidity, stilness, locking of the jaws. The blood becomes black. This is also the result of asphyxia, or of stragulation. Discoloration of the eyelids and protrusion of the tongue, would be indications of death from asphyxia, whether from strangulation or any other cause. All these appearances might exist without the adminstration of strychnine. I would consider strychnine the cause of death, if found in the stomach.

The mass as brought to me was of a brick-red color. Bread, cheese, and tomatoes were found in it. It was a pastry mass, weighing probably two and a half or three oz. of solid matter. A healthy man might have digested such a supper as Mrs. Green ate, in 4 or 6 hours. Strychnine in a medicinal dose, assists digestion. (Exhibits the two papers given to him by Dr. Bird.) One of these is marked "Quinine, 4 portions ?" I think I added the interrogation point myself. This paper contains 2½ or 3 grains, in powder—about a medicinal dose. I tested it by the new mode; the polarization of light. The other paper, marked Poison, in ink, I tested as before stated, and found it to be a heterogeneous mixture, apparently an adulterated specimen of strychnine. Brucine is almost always present in commercial strychnine. There are two kinds of strychnine known to druggists, the powdered and the crystals. This had the appearance of being composed of strychnine reduced to powder, and some other substance mixed with it. These are the papers I received from Dr. Bird. I have used about one-sixth of the quantity that I received, in experimenting and analyzing. The first taste of strychnine is extremely bitter; there is a second, indescribable, metallic taste, at the back of the throat, easily distinguishce by the experts. This taste would be produced in every case where strychnine had been swallowed to the extent of ⅛th of a grain. The first taste of strychnine is somewhat like the bitter of hops. This taste might be disguised in ale or porter; but the second back taste cannot thus be disguised.

I always administer strychnine in solution. I used to mix it with starch in powder. I have never directed a patient to procure strychnine for himself, and will never do so. When I first got the stomach &c. from Dr. Bird, I placed them in a cupboard which I kept locked, and never left the key out of my possession. I was careful to place all the articles in peculiar positions, so that I could easily tell if they had been moved. When I took them to the Medical College, I observed the same precautions, and in addition kept them under seal. The door of the cupboard, the windows, and the door of the room were all sealed with two seals each. The seals remained unbroken, except in one instance where one seal on a door became loose, though the other seal on the same door remained unbroken. No one except Mr. Butler had access to this room. Except the man who made the fires in my presence, no one else entered the room.

Death from cholera would not produce any of the appearances that have been described in the case of Mrs. Green, except discoloration. The contraction of the muscles of the extremities might however have resulted from the cramps of cholera.

Cross examined—I stopped giving strychnine in powder, because some of my patients did not observe my direction. One man, with whom I left one grain of strychine, divided into eight powders, took one, and finding that it produced no immediate apparent effect, took immediately the other seven. He nearly died, but came too again. In the administration of strychnine as a medicine, I always state what it is, and enjoin the utmost care in its use. Most of the powdered strychnine sold is adulterated; the crystals, not so much so. There is not near so much adulteration in drugs now, as formerly. The medical profession, and also the Pharmaceutical, have taken such strong grounds against the practice, that it has greatly abated. I chemically determined the presence of strychnine in the paper, marked *poison*, but could not say what other substance it contained. It was not more adulterated than the commercial strychnine usually is. Dr. Bird brought most of the article to me at one time. My investigations would have been the same, had I not suspected the existence of strychnine. I first looked for an alkaloid in the stomach. Subsequent tests were used to discover strychnine. At the Coroner's inquest my attention was directed to a class of poisons that would produce death without vomiting. The post mortem examination did not suggest arsenic to my mind. The finding of the papers directed my attention to the propriety of searching for a vegetable alkali, although I would have done so had I not found these papers. I have read of a case where a dose of strychnine of a grain, twice a day, has been taken without fatal effects. The quantity which I took from the stomach was *probably* 1-20 of a grain. I have good reason to suppose there was more in the stomach; think I could swear so. I could not have got the whole quantity. Could not swear positively that there was 1-15 of a grain. The appearance of a body after death, caused by strychnine, would vary, owing to various circumstances. No definite conclusion could be arrived at as to the time the strychnine had been in the stomach.

Here the Court took a recess.

---

AFTERNOON SESSION.

*Dr. Blaney* cross-examined by defence—For intermittent fever, I should consider $\frac{1}{8}$ of a grain to an adult male, two or three times a day, a dose; to an adult female a somewhat smaller dose. I cannot state what were the causes of Mrs. Green's death, from the post mortem examination, further than a high probability that the deceased died of asphyxia; I could not swear as to the cause of her death; leaving out of view my discovery of strychnine in the stomach, I would not swear, from the post mortem examination that deceased came to her death from other than natural causes—stating at the same time the high probability that she died of asphyxia.

Q.—Would the existence of 1-15 of a grain of strychnine in the stomach of a deceased person, would that fact alone, authorize you to state that she died from the effect of that strychnine?

A.—No, sir; I consider I cannot swear positively to that which I do not absolutely know.

Q.—Those tests of strychnine by you may be classed under "taste, color, and form" under the microscope?

A.—Yes, sir. Strychnine does not always present the same form of chrystalization when produced under different circumstances, but it is a very common thing for substances to crystalize in *compatible* forms; I have never been able to measure the angle of the chrystals found. The form of chrystals, when taken by themselves, is not sufficient to base an opinion; no one test by itself is sufficient to establish the conclusion; an accumulation of probabilities is all chemists ever claim, though we have a moral certainty; do not consider the forms of the chrystals in this case as establishing the presence of strychnine; should regard the "bitter taste" of the substance analyzed in this case the weakest test of all; in this case I mainly rely upon the tests of color; my chrystals were broken, and I could not measure the angles, else I might have arrived nearer to absolute certainty.

Q.—Independent of the tests of color, would you swear to the presence of strychnine?

A.—No. The number of alkaloids is numerous; many not yet discovered, without doubt; "alkaloid" and "vegetable alkali" is the same thing; morphia, brucia, strychnia, narcia, quinia, are all alkaloids; there are various others; all these are of the same class; strychnine, quinia and morphia have the property in common of forming salts with acids, and are derivable from vegetable substances, all bitter, all white when pure in powder or chrystal; carbon, hydrogen, oxygen, and nitrogen, are the elements of all these; all these elements are found in the stomach of the human body.

Q.—How recently have discoveries of a vegetable alkali been made?

A.—We scarcely receive three or four medical journals without learning of a new alkaloid; these tests of color are characteristic of strychnine, but it is not yet established that there may be no alkaloids of which they are not also characteristic· But they are characteristic of no alkaloids now known save strychnine

Q.—Would you have been better satisfied in this case if you could have made more extensive researches?

A.—I would have been glad to have made more researches, to ascertain if there be a vegetable alkali in the *tomato* found in her stomach. *I could not swear that there was not a vegetable alkali in tomato.*

Q.—Are not tests once believed to have yielded certain results, sometimes found to have been erroneous?

A.—Yes; positive tests forever remain, but there are tests that prove unreliable.

Q.—If you should discover any other substance which would respond to the tests used, would it not be necessary to look for further tests?

A.—We should use the same tests, but look further for tests to distinguish between them.

In Nature the variety of substances are infinite; in the laboratory of nature, there are substances all around that produce these colors of which I have spoken in my report of this investigation; there are a great variety of substances, the combinations of which would produce the colors alluded to.

Q—Is Orfila considered as a high authority in medical science?

Ans—He stands among the first.

Q—Do you remember of any trial where a chemical analysis by Orfila proved erroneous?

Ans—I remember reading a case some eight years since in which some errors of analysis were found because of an insufficient quantity of material being submitted to examination.

The tests of color to which I have referred in my examination were first brought to my notice in 1853: Davy's test is the most recent; Lafortes was known before, so was the test by peroxide of lead; until within five or six years the distinctive characteristics of strychnine were upon taste, chrystalline form, &c. The process through which I passed in the examination of this case was tedious, but not complicated. The great care I used in my investigation was to prevent the possibility of the introduction of any substance containing the slightest presence of alkaloids; I did this that the prisoner might not unjustly suffer from a mistake of the chemist; without extraordinary precautions there would be danger of error; this is the first case where I have used the same process in the manner detailed now.

Direct resumed—My reasons for declaring that I believe the amount of strychnine found by me in the stomach of deceased was not the whole quantity taken, are several; all the contents were not submitted to analysis; it is supposed from the length of time believed to have intervened between taking the strychnine and death, that a portion of the strychnine must have been absorbed from the stomach before death—In a stomach that was full the amount of absorption would be less because of the dilatation, &c. I have never known strychnine administered for cholera or cholera morbus.

Q—Taking into consideration the post mortem examination, the fact that strychnine found in the stomach, and that corresponding poison was discovered in the house where Mrs. Green died, what is your opinion as a medical man of the cause of her death?

Question objected to.

Q—Taking into consideration the post mortem examination as detailed by the witnesses, the fact that strychnine was found in stomach, and that the corresponding poison was discovered in the house where Mrs. Green died, what is your opinion as a medical man of the cause of her death.

Question objected to by defence. Objection overruled. Counsel for defence except to the ruling of the court.

Ans—A high probability that she came to her death from the effects of strychnine.

Q—Are or are not medical men usually satisfied that death was caused by poison unless presence of poison is detected in the system by chemical analysis?

Objected to and objection sustained.

Q—Do medical men generally form opinions in relation to death by poison unless they have detected the presence of poison in the system?

Objected to and objection sustained.

Q—Would you be satisfied death was occasioned by poison unless on chemical analysis poison was found in the system?

A—Cases would be exceedingly rare; I consider a chemical analysis the most satisfactory test; taking all the tests I have resorted to in this case, I have no doubt death was caused by strychnine: There is no proof that strychnine is an irritant—it is a vegetable poison; brucia and strychnine are both extracted from the nux vomica, they are known as alkaloids; the irritant properties of nux vomica lie in other substances contained in it rather than strychnine; there has never been discovered a poison possessing precisely the same chemical properties as strychnine; in this investigation I have applied all the tests known to chemists, as far as my knowledge extends, except the measurement of the corners of the angles, which I was prevented from doing by the crystals being broken.

Q—Is there more difficulty in detecting the presence of strychnine than of other poisons?

A—It is much more difficult to detect the presence of vegetable than of mineral poisons; carbon, hydrogen, oxygen and nitrogen, are the constituents of the alkalies; the most active medicinal substances contain all four elements—strychnine contains all four.

Q—Do each of the chemical tests applied by you give distinct, independent and corroborative evidence of the presence of strychnine.

Objected to and sustained. (The objection was subsequently withdrawn.)

A—The tests except those of color were corroborative without being distinctive.

Q—Is there any other substance known to chemical science which gives the same color as strychnine does, on the application of either of the tests you applied?

A—None to my knowledge. Taylor on poisons is a work of high character in our profession; Orfila and Chitty (upon law) are also good authorities.

B. F. Butler called—I assisted Dr. Blaney in the investigations he has detailed here; I had access to the analysis and saw that every precaution was taken; while I was in the building all the doors were locked; the door of the room in which the analysis was carried on was locked on the inside, and I had the key.

Dr. Freer called—I assisted in the post mortem examination of Mrs Green; am demonstrator of anatomy in Rush Medical College; I noticed the general appearance of Mrs. G.; noticed a discoloration that is exceedingly common in dead bodies at that period; the eyelids were distended, and there was a general puffiness of the body: the brain, lungs and heart were healthy; the heart was very empty, as were the large vessels near; it is seldom we can examine those organs in the living body; suppose my experience is rather extensive; I saw no indications of cholera; if strychnine has been discovered in the stomach I should hesitate to ascribe death to strychnine; strychnine may have been given by medical men and might be found in the stomach.

